# Morphometry of cerebral arterial bifurcations harbouring aneurysms: a case-control study

**DOI:** 10.1186/s12883-022-02559-8

**Published:** 2022-02-10

**Authors:** K. Ćmiel-Smorzyk, E. Kawlewska, W. Wolański, A. Hebda, P. Ładziński, W. Kaspera

**Affiliations:** 1 Department of Neurosurgery, Medical University of Silesia, Regional Hospital, 41-200 Sosnowiec, Poland; 2 Department of Biomechatronics, Silesian University of Technology, Zabrze, Poland; 3 Maria Sklodowska-Curie National Research Institute of Oncology, Gliwice, Poland

**Keywords:** Intracranial aneurysm, Morphometry, Principle of minimum work, Aneurysm formation

## Abstract

**Background:**

Conclusions from studies evaluating vessel dimensions and their deviations from values resulting from the principle of minimum work (PMW) on the formation of intracranial aneurysms (IAs) are still inconclusive. Our study aimed to perform a morphometric analysis of cerebral arterial bifurcations harbouring aneurysms.

**Methods:**

The study comprised 147 patients with basilar artery (BA) and middle cerebral artery (MCA) aneurysms and 106 patients constituting the control group. The following morphometric parameters were evaluated: the radii of vessels forming the bifurcation, the junction exponent, the values of the bifurcation angles (Φ_1_ and Φ_2_ angles between the parent vessel trunk axis and the larger or smaller branches, respectively; α angle, the total bifurcation angle) and the difference between the predicted optimal and observed branch angles.

**Results:**

The analysed parameters for internal carotid artery (ICA) bifurcations were not significantly different among the groups. The MCA and BA bifurcation angles and the radii of the parent MCA and BA vessels with aneurysms were significantly higher than those of the control group. The differences between the predicted optimal and observed branch angles were significantly higher for BA and MCA bifurcations with aneurysms compared to the control group. The mean junction exponent for bifurcations in the circle of Willis (i.e., ICA and BA bifurcations, respectively) and MCA bifurcations with aneurysms was significantly lower than the theoretical optimum and did not significantly differ among the groups. In a multilevel multivariate logistic regression analysis, the branch angles and the radius from the parent vessel were significant independent predictors of the presence of an IA. The ROC analysis indicated that the α angle was the best performer in discriminating between aneurysmal and nonaneurysmal bifurcations.

**Conclusions:**

The dimensions of the arteries forming the circle of Willis do not follow the PMW. Deviation from the energetically optimum geometry for bifurcations beyond the circle of Willis (particularly, a larger radius of the parent artery and a wider total bifurcation angle) may lead to the formation of IAs. Further studies are warranted to investigate the significance of vessel dimensions and the bifurcation angle on the magnitude of shear stress in the walls of arterial bifurcations.

## Background

Intracranial aneurysm (IA) is a disease with a complex multifactorial aetiology. The results of epidemiological and genetic studies indicate a strong involvement of genetic and environmental factors [1, 2]. Experimental glass model studies, in vivo studies, and computational fluid dynamics (CFD) studies have shown that the increase in haemodynamic stress caused by the impact of blood flow on bifurcation is the main factor initiating the formation of an IAs. It is the main factor responsible for destructive remodelling of the arterial wall, characterised by disruption of the internal elastic lamina, loss of medial smooth muscle cells, reduced proliferation of smooth muscle cells, and loss of fibronectin [3, 4].

Recently, published data have shown that the abovementioned vascular segments are affected by complex haemodynamic forces, such as wall shear stress (WSS), the WSS gradient (WSSG), and temporal fluctuations in WSS [3–10]. IAs usually arise at bifurcations, where the vessels are exposed to the maximum impact of WSS [5, 6]. The WSS experienced at a bifurcation is dependent on its geometry, including the radii of all vessels involved and the bifurcation angle [11–13]. WSS is minimised when the relation between the vessel diameter and the bifurcation angle follows the optimality principle of minimum work (PMW) [14].

According to the PMW, the biological system expends energy to maintain circulation and metabolism, and its efficiency depends on maintaining normal continuity of blood flow with minimal energy expenditure, including saving losses resulting from the increase in WSS. Murray used the PMW to predict vessel diameters and bifurcation angles in a theoretical vascular tree that is ideal for minimising the energy essential for ensuring blood flow continuity [15, 16]. Murray’s theoretical assumptions about the structure of the vascular network were confirmed by angiographic studies demonstrating that the dimensions of various human blood vessels [17, 18], including intracranial arteries [19], were consistent with the theoretical optimal values imposed by the PMW.

Based on current reports, deviations in the geometry of intracranial arterial bifurcations predisposed to cerebral aneurysm development, which are different from PMW, may be a key determinant for the formation of aneurysms [14, 20, 21]. Nevertheless, the conclusions from these reports are still inconclusive. Therefore, we planned a case-control study involving the selection of patients with IAs and non-aneurysmal controls to determine selected morphometric parameters and to analyse their relationship to the PMW-derived optimal values.

## Methods

### Patient population

The study included 147 patients consecutively admitted to the Department of Neurosurgery, Regional Hospital in Sosnowiec, Medical University of Silesia, Poland, between June 2013 and June 2020. One hundred fifteen patients presented with an unruptured middle cerebral artery (MCA) aneurysm (22 men, 93 women, aged 28 to 79 years [58 ± 10; mean ± SD]), and 32 patients presented with an unruptured basilar artery (BA) aneurysm (11 men, 22 women, aged 33 to 77 years [60 ± 11; mean ± SD]), which was confirmed by three-dimensional computed tomography angiography (3D CTA). The control group consisted of 106 patients who were sex- and age-matched to both study groups, including 38 men and 98 women aged 28 to 79 years (56 ± 12; mean ± SD) in whom no pathology was found on 3D CTA.

The exclusion criteria were as follows: the presence of a central nervous system disorder other than an IA that could affect cerebral arterial blood flow (e.g., ischaemic stroke, intracerebral haemorrhage, subarachnoid haemorrhage), other severe systemic conditions (e.g., advanced malignancy, severe circulatory or multiorgan failure), the presence of haemodynamically significant morphological changes in the extracranial segment of the internal carotid artery (ICA), a family history of IA and pregnancy.

Patients and controls were referred for 3D CTA after conventional computed tomography (CT) to rule out the presence of an IA or as a medical check-up of the brain for minor symptoms such as headache or vertigo.

### CTA protocol

CTA was performed using a 64-channel multidetector CT scanner (GE Optima CT660, GE Healthcare, USA) with IV bolus administration of a nonionic contrast medium (Omnipaque 350, GE Healthcare, USA). The scanning parameters included collimation of 39.38 × 0.625 mm, a spiral pitch of 0.984, a tube voltage of 120 kV, a tube amperage of 450 mA, a 0.4 s rotation time, and a slice thickness of 0.625 mm. A total of 50 mL of contrast medium followed by 30 mL of saline solution was injected into an antecubital vein at a rate of 4–4.5 mL/sec using a power injection platform (LF OptiVantage DH, USA). CT scanning was triggered by using a Smart Prep protocol, with the region of interest placed in the common carotid artery. Image acquisition started 5 sec after the attenuation reached 100 HU. The scanning time was approximately 4.5–6.0 s.

### Morphometric analysis of intracranial arterial bifurcations

**Fig. 1:**
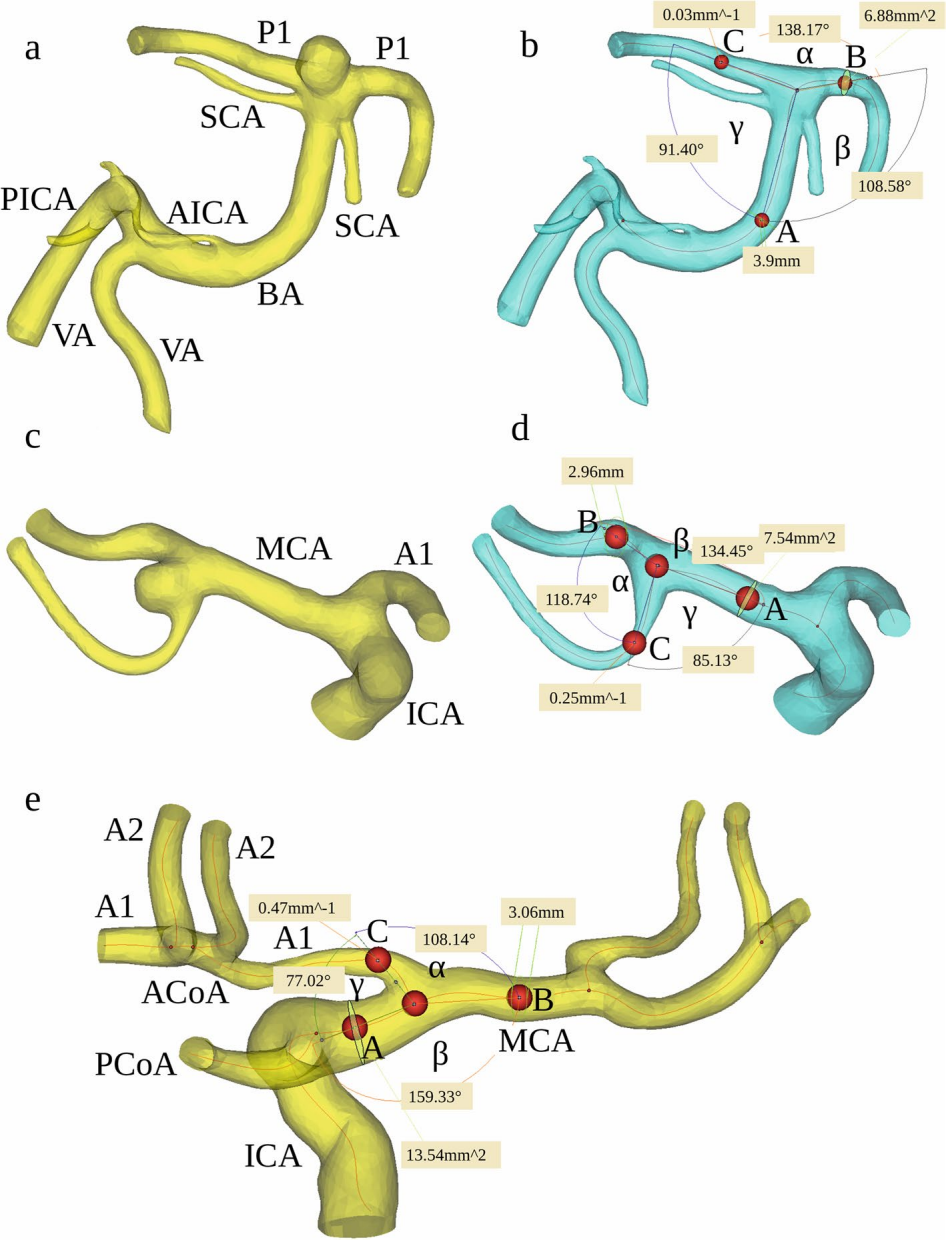
The three-dimensional model of the BA before (**a**) and after (**b**) digital removal of the aneurysm, the MCA before (**c**) and after (**d**) digital removal of the aneurysm, and the ICA – the control group – (**e**) obtained in Mimics v. 16.0 based on DICOM files from the CTA. The centreline (red line) was automatically fitted to the model. Points A, B and C correspond to the largest curvatures of the parent vessel of the BA, MCA or ICA and the postbifurcation branches (the larger and smaller branches, respectively); these are the points for which vessel cross-sectional areas and the best fit diameters were calculated automatically. The arms of the α angle were formed by points B and C and the point at the intersection of both centrelines. The β angle (between the trunk of the BA, MCA or ICA and the larger branch) was defined by points A and B and the point at the intersection of both centrelines. The γ angle (between the trunk of the BA, MCA or ICA trunk and the smaller branch) was defined by points A and C and the point at the intersection of both centrelines. BA, basilar artery; MCA, middle cerebral artery; ICA, internal carotid artery; A1, A1 segment of the anterior cerebral artery; A2, A2 segment of the anterior cerebral artery; ACoA, anterior communicating artery; PCoA, posterior communicating artery; P1, P1 segment of the posterior cerebral artery; SCA, superior cerebellar artery; AICA, anterior inferior cerebellar artery; PICA, posterior inferior cerebellar artery; VA, vertebral artery

The CTA data were transferred as DICOM files to a workstation equipped with the Mimics Innovation Suite platform (Materialise, Belgium). Mimics v. 16.0 and 3-matic v. 8.0 were used for image segmentation and the creation of a 3D vessel model. In all three groups of patients (patients with MCA aneurysms, patients with BA aneurysms, and control patients), the following were segmented: both ICAs with their bifurcations into the MCA and the anterior cerebral artery (ACA), the parent vessel of the MCA from the artery’s origin to its bifurcation and the postbifurcation branches, and the parent vessel of the BA with bifurcation into the posterior cerebral arteries (PCAs) (Fig. 1). Cases where the parent vessel of the MCA was divided into a trifurcation and cases that were not suitable for further morphometric calculations due to the poor quality of the 3D model were excluded from further morphometric analysis.

Finally, in the study group with MCA aneurysms, 119 MCA bifurcations with aneurysm (MCA bifs w/ An), 92 cases of opposite parent vessels of the MCA without aneurysm (MCA bifs w/o An), 108 bifurcations of the left ICA (L ICA), 107 bifurcations of the right ICA (R ICA), and 93 bifurcations of the BA were included in further morphometric analysis. In those patients with BA aneurysms, 23 bifurcations of the left MCA (L MCA), 23 bifurcations of the right MCA (R MCA), 29 bifurcations of the L ICA, 28 bifurcations of the R ICA, and 32 bifurcations of the BA with aneurysms (BA bifs w/ An) were included in the morphometric analysis. In the control group, 91 bifurcations of the L MCA, 91 bifurcations of the R MCA, 100 bifurcations of the L ICA, 99 bifurcations of the R ICA, and 91 bifurcations of the BA were included in the morphometric analysis.

In the groups of MCA and BA bifurcations with aneurysm, the aneurysm was digitally removed before morphometric analysis using the Mimics software, leaving the parent artery and the postbifurcation branches for further measurement. When the 3D models were finished, the vessel centreline was fitted automatically to each 3D model (Fig. 1) using a computer-aided design (CAD) tool. Using the centreline, the largest curvature of the parent vessels of the MCA, ICA and BA and the largest curvatures of each of the two post-bifurcation branches were automatically calculated. The points of the largest curvature were set as close to the bifurcation as possible but at a distance of at least 5 mm. Based on these points, the best fit diameter (d_0_) of the parent vessels of the MCA, ICA, and BA and the best fit diameter of both branches (d_1_ and d_2_ for the larger and smaller branches, respectively) were estimated automatically.

Next, the best fit diameters were used to calculate the radii of the parent vessels of the MCA, ICA, and BA (r_0_) and the radii of both branches (r_1_ and r_2_ for the larger and smaller branches, respectively) using the following formula:

1 $$\mathrm{r}=\mathrm{d}/2$$

where ‘r’ is the radius and ‘d’ is the best fit diameter. The radii were used to calculate two ratios, that is, the asymmetry ratio, which was calculated using the following formula:

2 $$\mathrm{asymmetry}\ \mathrm{ratio}={{\mathrm{r}}_2}^2{{\mathrm{r}}_1}^{-2}$$

and the area ratio calculated according to the formula:

3 $$\mathrm{area}\ \mathrm{ratio}=\left({{\mathrm{r}}_1}^2+{{\mathrm{r}}_2}^2\right){{\mathrm{r}}_0}^{-2}$$

Next, the centrelines with the largest curvature points were exported to 3-matic v. 8.0 to measure the angles between the bifurcation components. The points of the largest curvatures of the parent vessel and the larger and smaller branches and the point of the intersection of both centrelines were used to determine the apex of the angles.

The following angle values were calculated: the α angle between the branches of the MCA, ICA, and BA bifurcations, the β angle between the parent vessel of the vessel and the larger branch, and the γ angle between the parent vessel and the smaller branch (Fig. 1). Next, the angles between each of the two branches and the parent vessels of the MCA, ICA and BA axis were calculated:

4 $${\Phi}_1=180-\beta$$

5 $${\Phi}_2=180-\gamma$$

All morphometric measurements were performed by the same author (K. Ć-S).

### Calculation of predicted optimal morphometric parameters

According to the PMW, the adjustment of a given vascular system to its theoretical optimum is expressed as the junction exponent (n):

6 $${{\mathrm{r}}_0}^{\mathrm{n}}={{\mathrm{r}}_1}^{\mathrm{n}}+{{\mathrm{r}}_2}^{\mathrm{n}}$$

where the optimal value of the junction exponent for an energetically optimum construction of vessel bifurcation equals 3. The junction exponents for all MCA, ICA, and BA bifurcations were obtained with an online calculator available at http://www.wolframalpha.com.

The optimal angles between the axis of the parent vessels of the MCA, ICA and BA and the larger and smaller postbifurcation branches (ϕ_1_, ϕ_2_, respectively) as well as the total bifurcation angle (ϕ_1_ + ϕ_2_) were predicted using four PMW-derived optimality rules according to the following eqs. [22]:

7 $${\mathrm{cos}\upphi}_1=\left(\mathrm r_0^4+\mathrm r_1^4-\mathrm r_2^4\right)\left(2\mathrm r_0^2\mathrm r_1^2\right)^{-1}\xi\nu$$

8 $$\cos {\upphi}_2=\left({{\mathrm{r}}_0}^4+{{\mathrm{r}}_2}^4-{{\mathrm{r}}_1}^4\right){\left(2{{\mathrm{r}}_0}^2{{\mathrm{r}}_2}^2\right)}^{-1}$$

9 $$\cos\left({\upphi}_1+{\upphi}_2\right)=\left({\mathrm r}_0^4-{\mathrm r}_1^4-{\mathrm r}_2^4\right)\left(2{\mathrm r}_1^2{\mathrm r}_2^2\right)^{-1}$$

### Standard protocol approvals, registrations and patient consent

The present study’s protocol was approved by the Institutional Review Board at the Medical University of Silesia in Katowice, Poland, and all procedures were carried out in accordance with the relevant guidelines and regulations. Each patient was informed about the purpose and course of the research and asked to give their informed consent to participate in the project.

### Statistical analysis

Normal distribution of the study variables was verified with the Shapiro–Wilk test. The results are presented as the mean and standard deviation (SD). Values with a normal distribution were compared using one-way ANOVA, including post hoc analysis with the Tukey test for unequal sample sizes, and values with skewed distributions were compared using Kruskal–Wallis one-way ANOVA with multiple comparisons. All morphometric and haemodynamic parameters that showed significant intergroup differences were subjected to logistic regression analysis with a stepwise addition mode. The potential predictors of IA formation were identified using univariate analysis. Based on the univariate analysis, the variables with *p* values < 0.1 (except those correlated with one another) were included in the multivariate logistic regression model to identify the independent predictors of aneurysm formation. The results are presented as odds ratios (ORs) and 95% confidence intervals (CIs). The independent predictors of aneurysm formation were subjected to receiver operating characteristic (ROC) analysis to identify their cut-off values with optimal sensitivity and specificity. The Youden index was used to determine the optimal cut-off point. The results were considered statistically significant for *p* values < 0.05. Statistical analyses were performed with Statistica v. 13.3 (Tibco Software Inc.).

## Results

### Assessment of the statistical significance of the differences among the mean values of the morphometric variables for the ICA in the study groups

Table 1 shows a comparison of the morphometric variables, that is, the α, Φ_1_, and Φ_2_ angles, the differences between the predicted and observed values for the above angles, the radii (r_0_, r_1_, r_2_), the asymmetry ratio, the area ratio, and the junction exponent for the ICA among the patient groups (i.e., those with diagnosed MCA aneurysms, those with diagnosed BA aneurysms and the controls). No statistically significant differences were found in the mean values of the above variables among the study groups.

**Table 1:** Comparison of morphometric parameters for the ICA bifurcation among the patient groups

parameter	patients w/ MCA An		patients w/ BA An		control patients		*p* value
	R ICA	L ICA	R ICA	L ICA	R ICA	L ICA	
α angle (^o^)	116.4 ± 18.7	114.8 ± 15.4	119.4 ± 18.4	120.7 ± 26.4	120.2 ± 14.8	119.1 ± 14.7	NS
Φ_1_ angle (^o^)	44.9 ± 31.3	39.9 ± 30.3	35.6 ± 16.8	45.6 ± 19.2	41.7 ± 20.4	40.4 ± 20.6	NS
Φ_2_ angle (^o^)	88.2 ± 28.2	86.8 ± 27.4	96.0 ± 13.8	93.2 ± 15.6	95.0 ± 17.4	92.5 ± 19.1	NS
r_0_, mm	1.86 ± 0.24	1.85 ± 0.20	1.92 ± 0.23	1.91 ± 0.23	1.85 ± 0.23	1.82 ± 0.22	NS
r_1_, mm	1.48 ± 0.21	1.48 ± 0.18	1.53 ± 0.19	1.48 ± 0.23	1.49 ± 0.22	1.46 ± 0.23	NS
r_2_, mm	1.12 ± 0.22	1.13 ± 0.17	1.16 ± 0.21	1.17 ± 0.24	1.15 ± 0.25	1.13 ± 0.26	NS
asymmetry ratio, r_2_^2^r_1_^− 2^	0.59 ± 0.18	0.60 ± 0.17	0.59 ± 0.16	0.64 ± 0.20	0.62 ± 0.19	0.62 ± 0.20	NS
area ratio, (r_1_^2^+r_2_^2^)r_0_^− 2^	1.02 ± 0.21	1.03 ± 0.19	1.03 ± 0.23	1.00 ± 0.23	1.05 ± 0.21	1.04 ± 0.21	NS
n, junction exponent	2.28 ± 0.91	2.25 ± 0.78	2.38 ± 1.15	2.20 ± 0.78	2.40 ± 0.99	2.28 ± 0.76	NS
(ϕ_1_ + ϕ_2_) - α angle (^o^)	−57.2 ± 29.8	−59.5 ± 26.7	−60.3 ± 26.9	−65.4 ± 25.8	− 59.9 ± 29.2	−57.1 ± 28.5	NS
ϕ_1_ - Φ1 angle (^o^)	−23.4 ± 28.9	−25.7 ± 32.5	−13.0 ± 18.1	−18.8 ± 18.6	−19.0 ± 20.5	−19.4 ± 21.4	NS
ϕ_2_ - Φ2 angle (^o^)	−50.1 ± 32.3	−47.2 ± 34.4	−61.6 ± 20.0	−63.7 ± 21.3	−57.8 ± 23.1	−54.1 ± 24.5	NS

Continuous values are expressed as the mean ± SD. *An* aneurysm; *BA* basilar artery; *ICA* internal carotid artery; *MCA* middle cerebral artery; *R* right; *L* left; α angle, total bifurcation angle; Φ_1_ angle, angle between the axis of the parent vessel and the larger branch; Φ_2_ angle, angle between the axis of the parent vessel and the smaller branch; r_0_, radius of the parent vessel; r_1_, radius of the larger branch; r_2_, radius of the smaller branch; ϕ_1_ + ϕ_2_, total predicted optimal angle; ϕ_1_, predicted optimal angle between the axis of the parent vessel and the larger branch; ϕ_2_, predicted optimal angle between the axis of the parent vessel and the smaller branch

### Assessment of the statistical significance of the differences among the mean values of the morphometric variables for the MCA in the study groups

The results of the comparison of the morphometric variables for the MCA among the groups of patients with MCA aneurysms, patients with BA aneurysms, and control patients are given in Table 2. The post hoc analysis showed that the α, Φ_1_, and Φ_2_ angles in the MCA bifs w/ An group were significantly higher than the corresponding angles in the MCA bifs w/o An group and the control group. However, the α and Φ_2_ angles in the same bifurcation group were also significantly higher than the α and Φ_2_ angles of the R MCA and the L MCA in those patients with BA aneurysms.

**Table 2:** Comparison of the morphometric parameters for the MCA bifurcation among the patient groups

parameter	patients w/ MCA An		patients w/ BA An		control patients		*p* value
	MCA bifs w/ An	MCA bifs w/o An	R MCA	L MCA	R MCA	L MCA	
α angle (^o^)	128.2 ± 24.2	105.9 ± 19.1	103.4 ± 25.8	93.3 ± 18.0	97.1 ± 20.5	93.2 ± 19.6	< 0.001
Φ_1_ angle (^o^)	59.6 ± 25.2	47.3 ± 20.9	47.7 ± 23.0	49.9 ± 27.0	46.8 ± 23.1	42.4 ± 23.0	< 0.001
Φ_2_ angle (^o^)	81.5 ± 21.7	68.2 ± 20.4	66.4 ± 20.5	57.8 ± 25.2	61.0 ± 19.4	65.3 ± 18.2	< 0.001
r_0_, mm	1.40 ± 0.18	1.42 ± 0.18	1.54 ± 0.21	1.49 ± 0.25	1.35 ± 0.16	1.31 ± 0.20	< 0.001
r_1_, mm	1.16 ± 0.17	1.17 ± 0.19	1.24 ± 0.23	1.21 ± 0.23	1.15 ± 0.18	1.11 ± 0.21	NS
r_2_, mm	0.87 ± 0.20	0.91 ± 0.19	0.95 ± 0.22	0.94 ± 0.18	0.86 ± 0.17	0.84 ± 0.18	NS
asymmetry ratio, r_2_^2^r_1_^− 2^	0.60 ± 0.24	0.63 ± 0.21	0.56 ± 0.20	0.65 ± 0.17	0.59 ± 0.21	0.61 ± 0.20	NS
area ratio, (r_1_^2^+r_2_^2^)r_0_^− 2^	1.10 ± 0.20	1.11 ± 0.24	1.11 ± 0.17	1.10 ± 0.25	1.15 ± 0.21	1.15 ± 0.23	NS
n, junction exponent	2.49 ± 0.93	2.69 ± 1.19	2.56 ± 0.84	2.65 ± 1.20	2.84 ± 1.11	2.79 ± 1.06	NS
(ϕ_1_ + ϕ_2_) - α angle (^o^)	− 68.6 ± 29.7	−38.8 ± 30.5	−36.7 ± 34.1	−32.0 ± 24.7	− 28.2 ± 25.1	−30.0 ± 35.8	< 0.001
ϕ_1_ - Φ_1_ angle (^o^)	−38.6 ± 24.4	− 23.7 ± 21.0	− 26.4 ± 25.7	− 25.1 ± 21.8	− 23.1 ± 23.3	− 21.1 ± 26.6	< 0.001
ϕ_2_ - Φ_2_ angle (^o^)	−42.7 ± 27.3	−25.6 ± 22.8	− 24.0 ± 21.3	− 20.5 ± 27.1	− 16.0 ± 22.3	−23.3 ± 25.9	< 0.001

Bifs, bifurcations; see Table 1 for other abbreviations

Post hoc analysis:

α angle, MCA bifs w/ An significantly higher than MCA bifs w/o An*, R MCA (w/ BA An group)**, L MCA (w/ BA An group)*, R MCA (control group)* and L MCA (control group)*; MCA bifs w/o An significantly higher than L MCA (control group)**

Φ_1_ angle, MCA bifs w/ An significantly higher than MCA bifs w/o An**, R MCA (control group)** and L MCA (control group)*

Φ_2_ angle, MCA bifs w/ An significantly higher than MCA bifs w/o An*, R MCA (w/ BA An group)***, L MCA (w/ BA An group)*, R MCA (control group)* and L MCA (control group)*

r_0_, L MCA (control group) significantly smaller than MCA bifs w/ An**, MCA bifs w/o An**, R MCA (w/ BA An group)* and L MCA (w/ BA An group)***; R MCA (control group) significantly smaller than R MCA (w/ BA An group)*

(ϕ_1_ + ϕ_2_) - α angle, MCA bifs w/ An significantly lower than MCA bifs w/o An*, R MCA (w/ BA An group)***, L MCA (w/ BA An group)***, R MCA (control group)* and L MCA (control group)*

ϕ_1_ - Φ_1_ angle, MCA bifs w/ An significantly lower than MCA bifs w/o An**, R MCA (control group)** and L MCA (control group)*

ϕ_2_ - Φ_2_ angle, MCA bifs w/ An significantly lower than MCA bifs w/o An**, R MCA (control group)* and L MCA (control group)*

* *p* < 0.001, ** *p* < 0.01, *** *p* < 0.05

The radius (r_0_) of the parent MCA vessel in the groups of MCA bifs w/ An, MCA bifs w/o An and w/ BA An was larger than that of the parent MCA vessel in both control groups. It was also significantly larger than the L MCA in the control group. Furthermore, the R MCA in the control group was significantly smaller than the R MCA from the w/ BA An group.

The comparison of the differences between the predicted and observed values for the α, Φ_1_, and Φ_2_ angles of the MCA among the study groups showed that these differences were significantly greater for the α angle in the MCA w/ An group compared to all the other MCA bifurcations (i.e., MCA bifs w/o An, MCA bifurcation in the groups of patients with BA aneurysm and the controls). However, for the Φ_1_ and Φ_2_ angles, they were significantly greater than those of the MCA w/o An group and the control group.

### Assessment of the statistical significance of the differences among the mean values of the morphometric variables for the BA in the study groups

The results of the comparison of the morphometric variables for the BA among those patients with MCA aneurysms, BA aneurysms and the controls are given in Table 3. The post hoc analysis showed that the α, Φ1, and Φ2 angles and the r_0_ and r_1_ radii in the BA w/ An group were significantly higher than the variables in the other groups of BA bifurcations.

**Table 3:** Comparison of the morphometric parameters for the BA bifurcation among the patient groups

parameter	patients w/ MCA An	patients w/ BA An	control patients	*p* value
	BA	BA bifs w/ An	BA	
α angle (^o^)	107.37 ± 19.27	136.68 ± 22.98	107.02 ± 23.55	< 0.001
Φ_1_ angle (^o^)	54.60 ± 20.23	71.70 ± 18.95	55.73 ± 19.19	< 0.001
Φ_2_ angle (^o^)	60.51 ± 17.87	77.07 ± 24.00	58.23 ± 20.82	< 0.001
r_0_, mm	1.57 ± 0.22	1.76 ± 0.36	1.58 ± 0.25	< 0.05
r_1_, mm	1.17 ± 0.21	1.31 ± 0.25	1.12 ± 0.22	< 0.001
r_2_, mm	0.96 ± 0.24	1.01 ± 0.23	0.92 ± 0.27	NS
asymmetry ratio, r_2_^2^r_1_^− 2^	0.69 ± 0.21	0.62 ± 0.20	0.70 ± 0.25	NS
area ratio, (r_1_^2^+r_2_^2^)r_0_^−2^	0.94 ± 0.22	0.91 ± 0.23	0.90 ± 0.20	NS
n, junction exponent	1.98 ± 0.74	1.90 ± 0.68	1.83 ± 0.62	NS
(ϕ_1_ + ϕ_2_) - α angle (^o^)	− 57.9 ± 26.2	−87.2 ± 31.1	−50.4 ± 27.7	< 0.01
ϕ_1_ - Φ_1_ angle (^o^)	−30.2 ± 23.8	−49.2 ± 19.9	−30.5 ± 22.0	< 0.05
ϕ_2_ - Φ_2_ angle (^o^)	−33.2 ± 20.7	−48.4 ± 23.7	− 28.0 ± 19.5	NS

Bifs, bifurcations; see Table 1 for other abbreviations

Post hoc analysis

α angle, BA bifs w/ An significantly higher than BA from patients w/ MCA An*and BA from the control group*

Φ_1_ angle, BA bifs w/ An significantly higher than BA from patients w/ MCA An* and BA from the control group*

Φ_2_ angle, BA bifs w/ An significantly higher than BA from patients w/ MCA An* and BA from the control group*

r_0_, BA bifs w/ An significantly larger than BA from the control group***

r_1_, BA bifs w/ An significantly larger than BA from patients w/ MCA An*** and BA from the control group*

(ϕ_1_ + ϕ_2_) - α angle, BA bifs w/ An significantly lower than BA from patients w/ MCA An*** and BA from the control group**

ϕ_1_ - Φ_1_ angle, BA bifs w/ An significantly lower than BA from patients w/ MCA An***

* *p* < 0.001, ** *p* < 0.01, *** *p* < 0.05

The differences between the predicted and observed values for the α angle in the BA w/ An group were significantly greater than those in the other groups of BA bifurcations. However, for the Φ_1_ angle, they were significantly greater in the BA w/ An group than in the BA bifurcation group in those patients with MCA aneurysms.

### Comparison between the observed and predicted values of the α, Φ_1_, and Φ_2_ angles

Table 4 shows the comparison of the observed values of the α, Φ_1_, and Φ_2_ angles with their predicted values. In all study groups (i.e., the groups of patients with MCA aneurysms, patients with BA aneurysms, and control patients), the observed values of the above angles were significantly higher than their predicted values for all arterial bifurcations (i.e., ICA, MCA, and BA) (Table 4).

**Table 4:** Comparison of the observed vs. predicted α, Φ_1_ and Φ_2_ angles in the patient groups

	patients w/ MCA An		patients w/ BA An		control patients	
α angle (o)	R ICA	116.4 ± 18.7 vs. 59.5 ± 22.9*	R ICA	119.4 ± 18.4 vs. 58.9 ± 26.6*	R ICA	120.2 ± 14.8 vs. 62.3 ± 25.3*
	L ICA	114.8 ± 15.4 vs. 54.6 ± 24.9*	L ICA	120.7 ± 26.4 vs. 61.4 ± 19.3*	L ICA	119.1 ± 14.7 vs. 60.7 ± 24.9*
	MCA bifs w/ An	128.2 ± 24.2 vs. 62.2 ± 23.3*	R MCA	103.4 ± 25.8 vs. 66.6 ± 16.2**	R MCA	97.1 ± 20.5 vs. 71.2 ± 20.3*
	MCA bifs w/o An	105.9 ± 19.1 vs. 68.3 ± 24.9*	L MCA	93.3 ± 18.0 vs. 64.3 ± 25.8*	L MCA	93.2 ± 19.6 vs. 67.9 ± 26.3*
	BA	107.4 ± 19.3 vs. 48.5 ± 24.5*	BA	136.7 ± 23.0 vs. 52.9 ± 22.6**	BA	107.0 ± 23.6 vs. 63.0 ± 19.0*
Φ_1_ angle (^o^)	R ICA	44.9 ± 31.3 vs. 22.0 ± 8.3*	R ICA	35.6 ± 16.8 vs. 21.2 ± 10.6***	R ICA	41.7 ± 20.4 vs. 22.9 ± 10.2*
	L ICA	39.9 ± 30.3 vs. 19.3 ± 9.7*	L ICA	45.6 ± 19.2 vs. 22.9 ± 6.9**	L ICA	40.4 ± 20.6 vs. 21.5 ± 9.9*
	MCA bifs w/ An	59.6 ± 25.2 vs. 23.0 ± 10.4*	R MCA	47.7 ± 22.9 vs. 24.0 ± 7.9**	R MCA	46.8 ± 23.1 vs. 23.8 ± 9.2*
	MCA bifs w/o An	47.3 ± 20.9 vs. 24.3 ± 10.1*	L MCA	49.9 ± 27.0 vs. 25.0 ± 12.4*	L MCA	42.4 ± 23.0 vs. 23.3 ± 10.0*
	BA	54.6 ± 20.2 vs. 19.2 ± 9.7*	BA	71.7 ± 18.9 vs. 18.7 ± 7.7**	BA	55.7 ± 19.2 vs. 25.6 ± 9.5*
Φ_2_ angle (^o^)	R ICA	88.2 ± 28.2 vs. 37.5 ± 16.4*	R ICA	96.0 ± 13.8 vs. 37.7 ± 17.8*	R ICA	95.0 ± 17.4 vs. 39.3 ± 17.6*
	L ICA	86.8 ± 27.4 vs. 35.3 ± 17.1*	L ICA	93.2 ± 15.6 vs. 38.5 ± 15.3*	L ICA	92.5 ± 19.1 vs. 39.2 ± 19.3*
	MCA bifs w/ An	81.5 ± 21.7 vs. 39.2 ± 16.3*	R MCA	66.4 ± 20.5 vs. 42.6 ± 11.9**	R MCA	61.0 ± 19.4 vs. 47.4 ± 16.3*
	MCA bifs w/o An	68.2 ± 20.4 vs. 44.0 ± 19.8*	L MCA	57.8 ± 25.2 vs. 39.3 ± 14.3***	L MCA	65.3 ± 18.2 vs. 44.7 ± 21.4*
	BA	60.5 ± 17.9 vs. 29.2 ± 16.8*	BA	77.1 ± 24.0 vs. 34.3 ± 15.9**	BA	58.2 ± 20.8 vs. 37.4 ± 14.0*

For abbreviations, see Table 1. * *p* < 0.001, ** *p* < 0.01, *** *p* < 0.05

### Univariate and multivariate analyses

Univariate logistic regression analysis identified the r_0_; the junction exponent; the area ratio; the asymmetry ratio; the Φ_1_, Φ_2_, and α angles; and the Φ_1_, Φ_2_, and α angles classified in tertiles as significant prognostic factors for the formation of aneurysms (Table 5).

**Table 5:** Univariate analysis of all the analysed morphometric parameters

parameter	OR (95% CI)	*p* value
r_0_, mm	2.74 (1.24-6.08)	< 0.05
r_1_, mm	4.96 (1.80-13.62)	< 0.01
r_2_, mm	1.69 (0.67-4.28)	NS
Area ratio, (r_1_^2^+r_2_^2^)r_0_^−2^	0.89 (0.38-2.06)	NS
Asymmetry ratio, r_2_^2^ r_1_^−2^	0.57 (0.24-1.37)	NS
n, junction exponent	0.89 (0.73-1.09)	NS
α (°)	1.06 (1.04-1.07)	< 0.001
α (°)		
1st tertile (58.3-94.2)	1 (reference)	
2nd tertile (94.2-120.0)	4.57 (2.29-9.15)	< 0.001
3rd tertile (120.1-175.9)	22.01 (11.08-43.74)	< 0.001
Φ_1_ (°)	1.03 (1.02-1.04)	< 0.001
Φ_2_ (°)	1.05 (1.04-1.06)	< 0.001
Φ_1_ (°)		
1st tertile (1.3-41.3)	1 (reference)	
2nd tertile (41.3-63.8)	1.77 (1.05-3.02)	< 0.05
3rd tertile (63.8-165.9)	3.62 (2.16-6.05)	< 0.001
Φ_2_ (°)		
1st tertile (6.0–59.5)	1(reference)	
2nd tertile (59.5–77.6)	1.83 (1.02-3.28)	< 0.05
3rd tertile (77.6-117.3)	8.73 (4.99-15.30)	< 0.001

For abbreviations, see Table 1

Disregarding the correlated parameter and considering the parameters relevant to the univariate model, two multivariate logistic regression models were constructed. The first Model (A) included only the tertiles of the Φ_1_ and Φ_2_ angles and the r_0_ and r_1_ radii, while the second Model (B) included the tertiles of the α angle and the r_1_ and r_0_ radii. The final results of both analyses are given in Table 6.

**Table 6:** Final multivariate logistic regression analysis models (A, B) of the parameters associated with IA formation

A		
parameter	OR (95% CI)	*p* value
r_0_, mm	2.74 (1.09-6.89)	< 0.05
Φ_1_ (°)		
1st tertile (1.3-41.3)	1 (reference)	
2nd tertile (41.3-63.8)	2.53 (1.38-4.64)	< 0.01
3rd tertile (63.8-165.9)	5.09 (2.80-9.24)	< 0.001
Φ_2_ (°)		
1st tertile (6.0–59.5)	1 (reference)	
2nd tertile (59.5–77.6)	2.26 (1.23-4.18)	< 0.01
3rd tertile (77.6-117.3)	12.14 (6.58-22.41)	< 0.001
B		
α (°)		
1st tertile (58.3-94.2)	1 (reference)	
2nd tertile (94.2-120.0)	4.57 (2.29-9.15)	< 0.001
3rd tertile (120.1-175.9)	22.01 (11.08-43.74)	< 0.001

For abbreviations, see Table 1. The first Model (A) included only the tertiles of the Φ_1_ and Φ_2_ angles, r_0_ and r_1_; the second Model (B) included the tertiles of the α angle, r_0_ and r

In the first Model (A), the best predictors of aneurysm formation among the included variables were the radius of the parent vessel (r_0_) (OR = 2.74, 95% CI: 1.09–6.89, *p* < 0.05), the tertiles of the Φ_1_ angle (for the 2nd tertile, OR = 2.53, 95% CI: 1.38–4.64, *p* = 0 < 0.01; for the 3rd tertile, OR = 5.09, 95% CI: 2.80–9.24, *p* < 0.001), and the tertiles of the Φ_2_ angle (for the 2nd tertile, OR = 2.26, 95% CI: 1.23–4.18, *p* = 0.01; for the 3rd tertile, OR = 12.14, 95% CI: 6.58–22.41, p < 0.001).

In the second Model (B), the best predictors of aneurysm formation were the tertiles of the α angle (for the 2nd tertile, OR = 4.57, 95% CI: 2.29–9.15, p < 0.001; for the 3rd tertile, OR = 22.01, 95% CI: 11.08–43.74, p < 0.001) (Table 6).

### Predictors of aneurysm formation – the ROC analysis

**Fig. 2:**
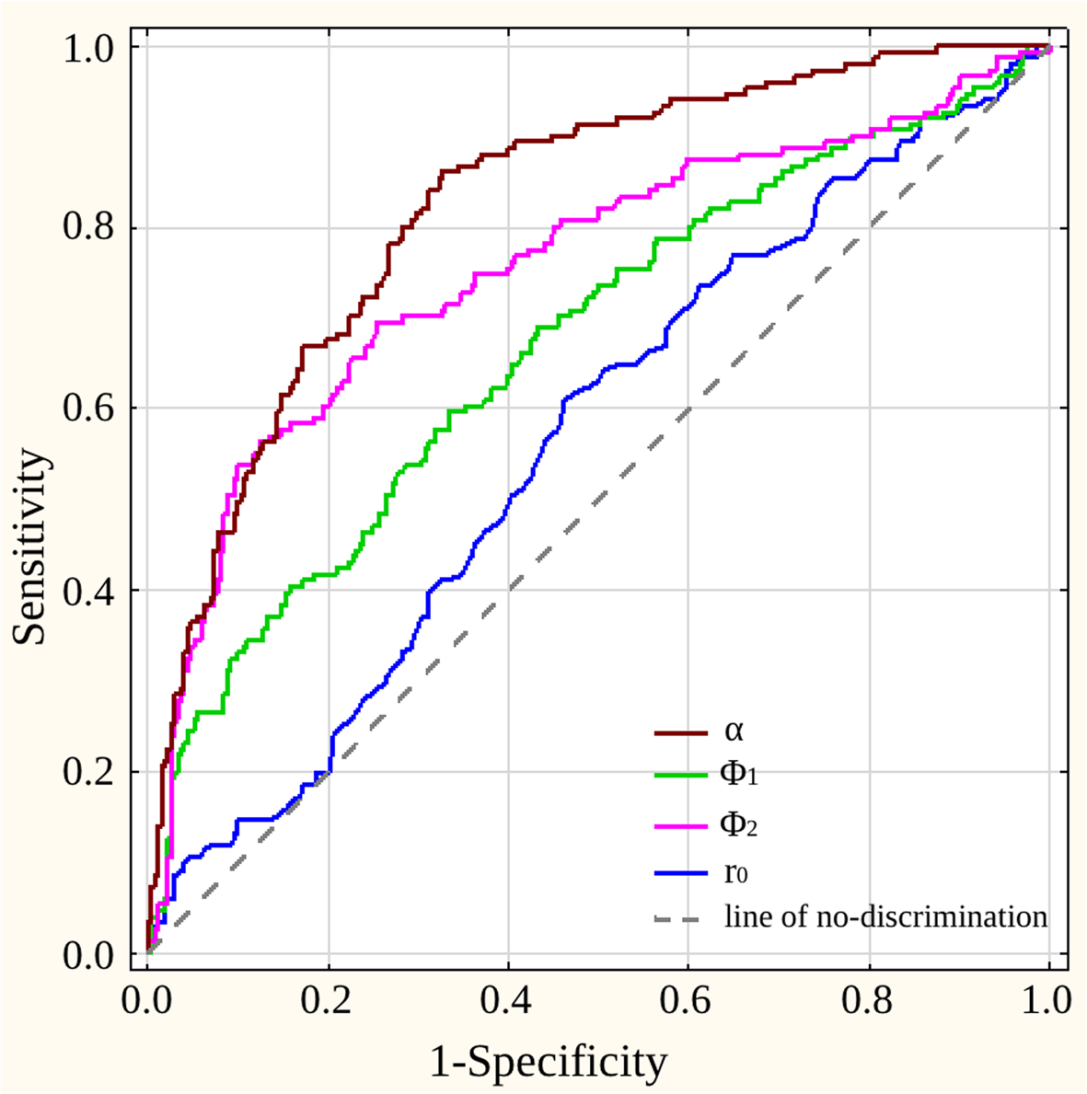
Receiver operator characteristic (ROC) curves for all the most significant predictors of aneurysm (description in the text). α angle, total bifurcation angle; Φ_1_ angle, angle between the axis of the parent vessel and the larger branch; Φ_2_ angle, angle between the axis of the parent vessel and the smaller branch; r_0_, radius of the parent vessel

The ROC curves for all the independent predictors for aneurysm formation are given in Fig. 2. The largest area under the curve (AUC) value was observed for the α angle (AUC = 0.826), followed by Φ_1_ (AUC = 0.670), Φ_2_ (AUC = 0.756), and r_0_ (AUC = 0.565), which implied that the α angle is the most accurate predictor of aneurysm formation among all the variables included in the logistic regression model. The optimal cut-off values for the Φ_1_, Φ_2_ and α angles and r_0_, which most accurately distinguished between aneurysmal and nonaneurysmal bifurcations, were 104.5° for the α angle (sensitivity of 0.86, specificity of 0.67), 58.8° for the Φ_1_ angle (sensitivity of 0.60, specificity of 0.67), 73.5° for the Φ_2_ angle (sensitivity of 0.70, specificity of 0.75) and 1.41 mm for the r_0_ (sensitivity of 0.61, specificity of 0.54).

## Discussion

The main findings of this study are as follows: (1) the deviation from the energetically optimum geometry for bifurcations beyond the circle of Willis (particularly, a larger radius of the parent artery and a wider total bifurcation angle) may lead to the formation of IAs, and (2) the dimensions of the arteries forming the circle of Willis do not follow the PMW.

According to the current view on the aetiology of IA, the haemodynamic factor, that is, mainly the magnitude of WSS and its gradient, plays a key role in the formation of aneurysms [10, 23].

The results of liquid flow in glass model studies and CFD studies have shown that the geometry of the bifurcation, including the diameter of the vessels forming the bifurcation and the bifurcation angle, plays an important role in the distribution of WSS and turbulence on the bifurcation components [12, 13, 21, 24–26]. For example, Roach et al., in their glass model study, showed that when the bifurcation angle increased, the risk of turbulence at the bifurcation apex also increased, which posed a risk of endothelial damage [12]. Using CFD simulations performed on parametric BA models, Tütüncü et al. showed that the change in the bifurcation angle from narrower to wider angles resulted in a significant widening of the area of accelerating WSS towards the daughter vessels [24]. In turn, using glass models of the anterior communicating artery (ACoA) complex, Ujiie et al. showed that when the asymmetry of the A1 segments of both ACAs increased or the flow in one of the two A1 segments increased, a significant increase was reported in WSS (above 70 Pa) exerted on the wall of the ACoA [13]. Additionally, CFD studies also showed that an increase in the asymmetry of the A1 segments of ACAs resulted in a significant increase in WSS (above 30 Pa) in the region of the dominant A1 segment/ACoA bifurcation [25].

The results of these studies are reflected in the results of observations of vascular anomalies in humans. It was clearly shown that the presence of asymmetry of the A1 segments of the ACoA complex was significantly associated with the formation of cerebral aneurysms at the junction of the dominant A1 segment and the ACoA [27–29]. According to Stehbens, this is due to the increased blood flow in the vessel with a larger radius, which causes an increase in haemodynamic stress [30].

However, the conclusions from the morphometric analysis of patient-derived models of the arteries of the circle of Willis with IAs are not always conclusive. Our study results showed that in patients with MCA and BA aneurysms, the radii of the parent vessels were significantly larger than those of the control group. Moreover, the parent vessel diameter was also one of the independent factors associated with the occurrence of IA. These results are consistent with our previous report in which we showed that the increase in the diameter of the parent MCA vessel (and hence the increase in the cross-sectional area of the parent vessel) resulted in a significant increase in the volume flow rate (VFR) that initiated aneurysm formation by increasing WSS at the bifurcation [31]. We also showed that VFR was a factor independently associated with the formation of MCA aneurysms [31]. However, the ROC analysis in the present study showed that the radius of the parent vessel was a poor predictor of IA formation (Tables 5 and 6; Fig. 2). Nevertheless, Can et al. demonstrated that the presence of BA and MCA aneurysms was significantly associated with a smaller radius of the parent vessel compared to that in the control group [32, 33]. According to those researchers, when the cross-sectional area of the parent vessel decreases, the blood flow velocity increases, resulting in a region of maximum haemodynamic stress at the apex of the bifurcation [32–34].

In the aetiology of IA, next to the parent vessel diameter, the symmetry of the primary bifurcation branches plays a key role. Many reports have shown that the greater the asymmetry of the branches forming the cerebral arterial bifurcation, the higher the risk of aneurysm formation [20, 21, 27, 31, 35–40]. According to Zhang et al., asymmetric bifurcation of the vessel increases the risk of aneurysm formation through the possible induction of abnormally enhanced haemodynamic stresses in the bifurcation [39, 40].

Some authors believe that the significance of the symmetry of bifurcation vessels for the formation of aneurysms cannot be considered without the theoretical assumptions of the PMW [14, 19, 20, 31]. According to the PMW, continuous blood flow in the vascular system is achieved with the minimal expenditure of energy to maintain it, including saving losses resulting from the increase in WSS. According to the PMW, a balance between energy dissipation due to frictional resistance of laminar flow (shear stress) and the minimum volume of the blood and vessel wall tissue is achieved when the vessel radii are adjusted to the cube root of the volumetric flow (formula no. 6) [15, 16]. Therefore, from a theoretical perspective, the adjustment of a given vascular system to its energetic optimum is expressed as the junction exponent (n) in the above equation. If the radii of bifurcation vessels fulfil Murray’s formula with *n* = 3, the energy expenditure for circulation maintenance and the magnitude of WSS are the lowest, regardless of the bifurcation asymmetry [14].

Our study showed that the values of indices that determine the symmetry of the MCA and BA bifurcations were not significantly different among the study groups (Tables 2 and 3). Nevertheless, in the MCA bifurcations with aneurysms, the value of the junction exponent (n) was significantly lower than that in the other groups (Table 2). This means that the vascular dimensions of the MCA bifurcation with an aneurysm do not follow the PMW, which could result in higher haemodynamic stress in MCA bifurcations and the formation of an aneurysm. These results are in line with the findings of other authors who also reported deviations in the value of the junction exponent (n) from n = 3 in bifurcations with aneurysms [14, 20, 21]. Of note, the other two bifurcations (i.e., ICA and BA) were characterised by junction exponent (n) values significantly deviating from 3 in all study groups (Tables 1 and 3). This finding is in line with the observations of Ingebrigtsen et al., who found that the values of the junction exponent (n) were significantly lower for ICA and BA bifurcations (both with and without an aneurysm).

According to Ingebrigtsen et al., the PMW establishes strict functional relations between volumetric flow, flow velocity, and the vessel dimensions and bifurcation angles of a typical vascular tree in which there is no communication between the bifurcation branches. However, blood flow through the circle of Willis includes the combination of flow from three vessels (both ICAs and the BA) further communicated through the communicating arteries. Therefore, the unique anatomy of the circle of Willis, significantly different from the normal branching nature addressed by the optimality principle, results in the fact that the arterial bifurcations of the circle of Willis do not follow the PMW. According to Ingebrigtsen et al., bifurcations in the circle of Willis may be consistent with the optimality principles that have not yet been determined [41]. While the MCA bifurcation and other bifurcations of cerebral arteries beyond the circle of Willis follow the optimality principle, the formation of aneurysms is associated with deviations from the optimal bifurcation geometry [41].

Most imaging-based studies (e.g., 3D rotational angiography, MRA, CTA) have shown that a wide bifurcation angle constitutes a significant risk factor for IA formation. Those studies included bifurcations that were generally considered to be predisposed to aneurysm development: the ACoA complex [27, 37, 40, 42–44], the BA [24, 33, 38, 40], and the MCA [31, 32, 39, 40, 45].

We also found that BA and MCA bifurcation angles in those patients with BA and MCA aneurysms were significantly higher than the other BA and MCA bifurcation angles used for the comparison.

Furthermore, the total bifurcation angle was the best predictor for the risk assessment for cerebral aneurysm formation (univariate and multivariate analyses, Fig. 2). To date, only a few studies that have evaluated the effect of the bifurcation angle on the magnitude of shear stress at vessel bifurcations using CFD simulations have shown that an increase in the total bifurcation angle results in abnormally enhanced haemodynamic stresses at the arterial bifurcations [24, 26, 42].

On the other hand, we found that the value of the total angles of ICA, BA, and MCA bifurcations without an aneurysm was significantly different from the values predicted by the PMW. The above discrepancies become understandable in light of the results of Zamir and Bigelow, who reported that even considerable deviations from the optimal angles could result in a relatively low (2–5%) increase in energy cost [22, 46]. Nevertheless, the differences between the predicted and observed values in the bifurcation groups were greater in the groups of MCA and BA bifurcations with an IA compared to BA and MCA bifurcations without an aneurysm. These results are in line with Ingebrigtsen et al., who analysed 107 BA, ICA, and MCA bifurcations with and without aneurysm and found significant differences among groups with respect to the mean bifurcation angles and the mean differences between the predicted optimal and observed angles [41].

### Limitations

Our study has several limitations. First, this investigation may have been affected by selection bias, which is one of the major drawbacks of case-control studies. We attempted to limit the influence of bias on the selection of patients into both groups (i.e., controls and the study group) by adopting a prospective nature of their selection into the above groups based on the previously established inclusion and exclusion criteria and by matching both groups in terms of sex and age. The use of exclusion criteria was aimed at eliminating potential confounders, such as diseases affecting cerebral circulation or a family history of intracranial aneurysms. However, given the retrospective nature of the study, we cannot exclude the possibility that the control group might have included patients who may develop an aneurysm later in life. Second, given the retrospective nature of our study, we must consider the possibility that the bifurcation morphology, including the bifurcation angles, may have changed after aneurysm formation. Third, although participants were recruited prospectively, some patients with aneurysms that were not detected on CT because of their small size may have been inadvertently excluded. Fourth, since only three selected bifurcations in the circle of Willis were analysed, further studies are warranted to evaluate the morphology of a large number of bifurcations in the circle of Willis and other bifurcations of intracranial arteries beyond the circle of Willis. Finally, further studies using CFD techniques are warranted to assess the changes in shear stress values that could verify the relationship of deviations in bifurcation vessel dimensions and bifurcation angle values to aneurysm formation.

## Conclusions

The dimensions of the arteries of the circle of Willis (both ICAs and the BA) do not follow the PMW, whereas the vascular dimensions of the cerebral arteries beyond the circle of Willis (e.g., the MCA) are governed by the optimality principle. Deviations in the dimensions of the bifurcation vessels of the circle of Willis and the arteries beyond the circle of Willis (particularly, a wider parent vessel diameter of the bifurcation and a wider total angle of the bifurcation) may lead to IA formation. In addition, in the case of arteries beyond the circle of Willis, deviations in the symmetry of the bifurcation that do not follow the optimality principle may also result in the formation of IAs. Further studies are warranted to investigate the significance of vessel dimensions and the bifurcation angle with respect to the magnitude of shear stress in bifurcation vessels.

## Data Availability

Fully anonymized data not published within this article will be made available by request from any qualified investigator following the EU General Data Protection Regulation.

## Abbreviations

3D CTA: Three-dimensional computed tomography angiography
ACA: Anterior cerebral artery
ACoA: Anterior communicating artery
AICA: Anterior inferior cerebellar artery
AUC: Area under the curve
BA: Basilar artery
CAD: Computer-aided design
CFD: Computational fluid dynamics
CI: Confidence interval
CTA: Computed tomography angiography
IA: Intracranial aneurysm
ICA: Internal carotid artery
MCA: Middle cerebral artery
MRA: Magnetic resonance angiography
OR: Odds ratio
PCoA: Posterior communicating artery
PICA: Posterior inferior cerebellar artery
PMW: Principle of minimum work
ROC: Receiver operating characteristic
SCA: Superior cerebellar artery
SD: Standard deviation
VA: Vertebral artery
VFR: Volume flow rate
WSS: Wall shear stress
WSSG: Wall shear stress gradient
